# 4-Hy­droxy­anilinium 2-carb­oxy­acetate

**DOI:** 10.1107/S160053681202363X

**Published:** 2012-05-31

**Authors:** Ying-Chun Wang

**Affiliations:** a College of Chemistry and Chemical Engineering, Southeast University, Nanjing 210096, People’s Republic of China

## Abstract

In the title compound, C_6_H_8_NO^+^·C_3_H_3_O_4_^−^, the amino N atom is protonated, and one of the carboxyl groups is deprotonated to maintain the charge balance. In the crystal, classical N—H⋯O and O—H⋯O hydrogen bonds connect the ions into a two-dimensional network parallel to the *ac* plane. In addition, the structure is further stabilized by C—H⋯O and π–π inter­actions [centroid–centroid distance = 4.115 (2) Å].

## Related literature

For the structures and properties of related compounds, see: Chen *et al.* (2001 ▶); Wang *et al.* (2002 ▶); Xue *et al.* (2002 ▶); Huang *et al.* (1999 ▶); Zhang *et al.* (2001 ▶); Ye *et al.* (2008 ▶).
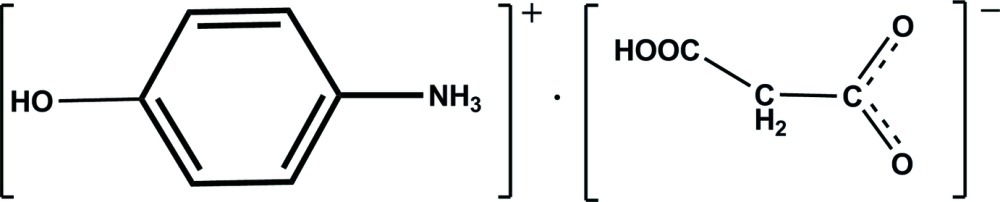


## Experimental

### 

#### Crystal data

C_6_H_8_NO^+^·C_3_H_3_O_4_^−^
*M* *_r_* = 213.19
Monoclinic, 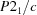

*a* = 5.1416 (1) Å
*b* = 22.5507 (7) Å
*c* = 7.8176 (3) Å
β = 97.827 (1)°
*V* = 897.98 (5) Å^3^
*Z* = 4
Mo *K*α radiation
μ = 0.13 mm^−1^
*T* = 173 K
0.10 × 0.05 × 0.05 mm

#### Data collection

Rigaku Mercury CCD diffractometer
Absorption correction: multi-scan (*CrystalClear*; Rigaku, 2005 ▶) *T*_min_ = 0.910, *T*_max_ = 1.000
6380 measured reflections
2040 independent reflections
1611 reflections with *I* > 2σ(*I*)
*R*_int_ = 0.041

#### Refinement

*R*[*F* ^2^ > 2σ(*F* ^2^)] = 0.050
*wR*(*F* ^2^) = 0.133
*S* = 1.07
2040 reflections
137 parameters
5 restraints
H-atom parameters constrained
Δρ_max_ = 0.31 e Å^−3^
Δρ_min_ = −0.40 e Å^−3^

### 

Data collection: *CrystalClear* (Rigaku, 2005 ▶); cell refinement: *CrystalClear*; data reduction: *CrystalClear*; program(s) used to solve structure: *SHELXS97* (Sheldrick, 2008 ▶); program(s) used to refine structure: *SHELXL97* (Sheldrick, 2008 ▶); molecular graphics: *SHELXTL* (Sheldrick, 2008 ▶); software used to prepare material for publication: *SHELXTL*.

## Supplementary Material

Crystal structure: contains datablock(s) I, global. DOI: 10.1107/S160053681202363X/rk2357sup1.cif

Structure factors: contains datablock(s) I. DOI: 10.1107/S160053681202363X/rk2357Isup2.hkl

Supplementary material file. DOI: 10.1107/S160053681202363X/rk2357Isup3.cml

Additional supplementary materials:  crystallographic information; 3D view; checkCIF report

## Figures and Tables

**Table 1:** Hydrogen-bond geometry (Å, °)

*D*—H⋯*A*	*D*—H	H⋯*A*	*D*⋯*A*	*D*—H⋯*A*
O5—H5⋯O3^i^	0.82	1.94	2.745 (2)	168
N1—H1*A*⋯O2^ii^	0.89	2.10	2.989 (2)	177
N1—H1*B*⋯O4^iii^	0.89	2.33	3.090 (2)	144
N1—H1*C*⋯O2^iv^	0.89	1.98	2.836 (2)	160
O1—H1⋯O4	0.82	1.65	2.450 (2)	165
C3—H3*A*⋯O3^iii^	0.93	2.47	3.398 (3)	175
C8—H8*B*⋯O1^v^	0.97	2.31	3.163 (3)	147

Symmetry codes: (i) 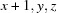; (ii) 
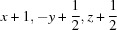
; (iii) 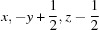; (iv) 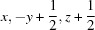; (v) 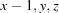.

**Fig. 1.**
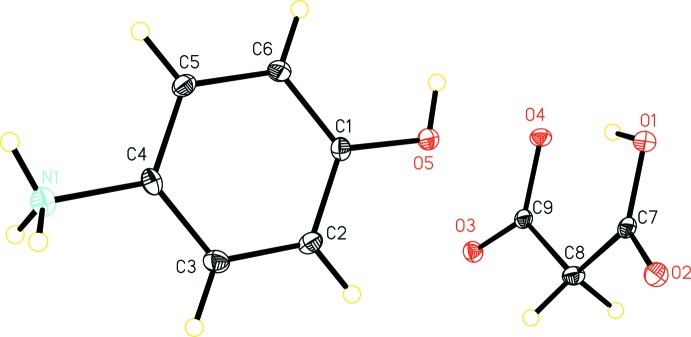
A view of the asymmetric unit with the atomic numbering scheme. The displacement ellipsoids were drawn at the 30% probability level. H atoms are presented as a small spheres of arbitrary radius.

**Fig. 2.**
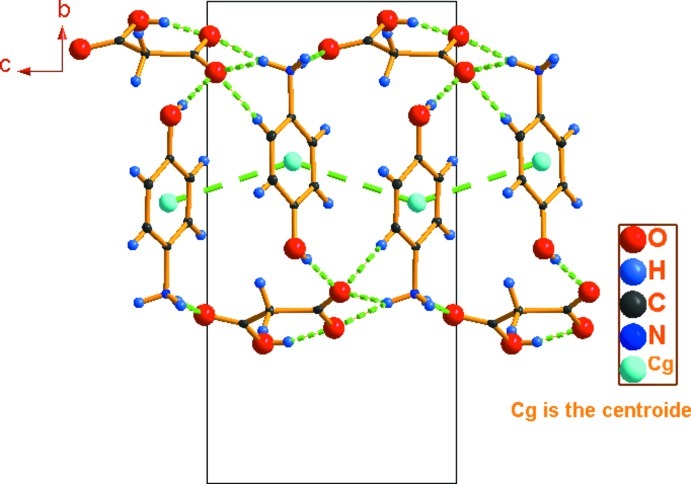
The crystal packing of the title compound viewed along the a axis showing the H-bonding and π–π interactions (dotted line).

## supplementary crystallographic information

### Comment

Simple organic salts containing strong intrermolecular H-bonds have attracted an attention as materials which display ferroelectric-paraelectric phase transitions (Chen *et al.*, 2001; Huang *et al.*, 1999; Zhang *et al.*, 2001). With the purpose of obtaining phase transition crystals of organic salts, various organic molecules have been studied and a series of new crystal materials have been elaborated (Wang *et al.*, 2002; Xue *et al.*, 2002; Ye *et al.* ,2008). Herewith, we present the synthesis and crystal structure of the title compound, 4-hydroxyanilinium 2-carboxyacetate.

In the title compound (Fig. 1), the bond lengths and angles have normal values. The asymmetric unit was composed of one 4-hydroxyanilinium cation and one 2-carboxyacetate anion. The protonated N atom was involved in strong intramolecular N–H···O hydrogen bonds with the N···O distances of N1–H1A···O2^ii^ - 2.989 (2)Å; N1–H1B···O4^iii^ - 3.090 (2)Å and N1–H1C···O2^iv^ - 2.836 (2)Å. The N–H···O and O–H···O H-bonding interactions connected the ion units into a 2D network parallel to the *ac*-plane. The weak non-classical intermolecular C3–H3A···O3^iii^ and C8–H8B···O1^v^ interactions were presented in the crystal structure with C3···O3^iii^ = 3.398 (3)Å and C8···O1^v^ = 3.163 (3)Å, respectively. The crystal packing was further stabilized by aromatic π–π interactions between the benzene rings of the neighbouring cations with the Cg···Cg distances of 4.115 (2)Å (Cg is the centroide of the benzene ring) (Fig. 2 and Table 1). Symmetry codes: (ii) *x*+1, -*y*+1/2, *z*+1/2; (iii) *x*, -*y*+1/2, *z*-1/2; (iv) *x*, -*y*+1/2, *z*+1/2, (v) *x*-1, *y*, *z*.

### Experimental

The malonic acid (10 mmol), 4-aminophenol (10 mmol) and ethanol (50 mL) were added into a 100 mL flask. The mixture was stirred at 333 K for 2 h, and then the precipitate was filtrated out. Colourless crystals suitable for X-ray diffraction were obtained by slow evaporation of the solution.

### Refinement

All the H atoms attached to C atoms were placed into the idealized positions and treated as riding with C–H = 0.93Å (aromatic) and C–H = 0.97Å (methylene) with *U_iso_*(H) = 1.2*U_eq_*(C). The H atoms based on N and O were placed into the calculated positions with the H–N = 0.89Å and H–O = 0.82Å and refined with *U_iso_*(H) = 1.5*U_eq_*(N and O).

### Crystal data

**Table:**

C_6_H_8_NO^+^·C_3_H_3_O_4_^−^	*F*(000) = 448
*M**_r_* = 213.19	*D*_x_ = 1.577 Mg m^−^^3^
Monoclinic, *P*2_1_/*c*	Mo *K*α radiation, λ = 0.71073 Å
Hall symbol: -P 2ybc	Cell parameters from 2040 reflections
*a* = 5.1416 (1) Å	θ = 2.8–27.5°
*b* = 22.5507 (7) Å	µ = 0.13 mm^−^^1^
*c* = 7.8176 (3) Å	*T* = 173 K
β = 97.827 (1)°	Block, colourless
*V* = 897.98 (5) Å^3^	0.10 × 0.05 × 0.05 mm
*Z* = 4	

### Data collection

**Table:**

Rigaku Mercury CCD diffractometer	2040 independent reflections
Radiation source: fine-focus sealed tube	1611 reflections with *I* > 2σ(*I*)
Graphite monochromator	*R*_int_ = 0.041
Detector resolution: 13.6612 pixels mm^-1^	θ_max_ = 27.5°, θ_min_ = 2.8°
CCD profile fitting scans	*h* = −5→6
Absorption correction: multi-scan (*CrystalClear*; Rigaku, 2005)	*k* = −28→27
*T*_min_ = 0.910, *T*_max_ = 1.000	*l* = −10→10
6380 measured reflections	

### Refinement

**Table:**

Refinement on *F*^2^	Primary atom site location: structure-invariant direct methods
Least-squares matrix: full	Secondary atom site location: difference Fourier map
*R*[*F*^2^ > 2σ(*F*^2^)] = 0.050	Hydrogen site location: inferred from neighbouring sites
*wR*(*F*^2^) = 0.133	H-atom parameters constrained
*S* = 1.07	*w* = 1/[σ^2^(*F*_o_^2^) + (0.0634*P*)^2^ + 0.3433*P*] where *P* = (*F*_o_^2^ + 2*F*_c_^2^)/3
2040 reflections	(Δ/σ)_max_ < 0.001
137 parameters	Δρ_max_ = 0.31 e Å^−^^3^
5 restraints	Δρ_min_ = −0.40 e Å^−^^3^

### Special details

**Table:**

Geometry. All s.u.'s (except the s.u. in the dihedral angle between two l.s. planes) are estimated using the full covariance matrix. The cell s.u.'s are taken into account individually in the estimation of s.u.'s in distances, angles and torsion angles; correlations between s.u.'s in cell parameters are only used when they are defined by crystal symmetry. An approximate (isotropic) treatment of cell s.u.'s is used for estimating s.u.'s involving l.s. planes.
Refinement. Refinement of *F*^2^ against ALL reflections. The weighted *R*-factor w*R* and goodness of fit *S* are based on *F*^2^, conventional *R*-factors *R* are based on *F*, with *F* set to zero for negative *F*^2^. The threshold expression of *F*^2^ > 2σ(*F*^2^) is used only for calculating *R*-factors(gt) etc. and is not relevant to the choice of reflections for refinement. *R*-factors based on *F*^2^ are statistically about twice as large as those based on *F*, and *R*-factors based on ALL data will be even larger.

### Fractional atomic coordinates and isotropic or equivalent isotropic displacement parameters (Å^2^)

**Table:**

	*x*	*y*	*z*	*U*_iso_*/*U*_eq_	
O5	0.4476 (3)	0.34202 (6)	0.35078 (19)	0.0191 (3)	
H5	0.5845	0.3567	0.3982	0.029*	
O3	−0.1396 (3)	0.40201 (6)	0.53276 (17)	0.0187 (3)	
O2	0.1261 (3)	0.43371 (6)	−0.01270 (17)	0.0183 (3)	
O1	0.3518 (3)	0.47345 (6)	0.22298 (18)	0.0184 (3)	
H1	0.3277	0.4710	0.3244	0.028*	
O4	0.2284 (3)	0.45183 (6)	0.50832 (17)	0.0182 (3)	
C4	0.5512 (4)	0.16064 (8)	0.3365 (2)	0.0155 (4)	
C3	0.3222 (4)	0.18648 (10)	0.2564 (3)	0.0193 (4)	
H3A	0.1887	0.1630	0.1995	0.023*	
N1	0.5772 (4)	0.09560 (7)	0.3326 (2)	0.0193 (4)	
H1A	0.7383	0.0853	0.3796	0.029*	
H1B	0.5489	0.0830	0.2237	0.029*	
H1C	0.4601	0.0792	0.3921	0.029*	
C8	−0.0615 (4)	0.42836 (9)	0.2494 (2)	0.0171 (4)	
H8A	−0.1252	0.3894	0.2111	0.021*	
H8B	−0.2060	0.4559	0.2224	0.021*	
C9	0.0095 (4)	0.42644 (8)	0.4436 (2)	0.0152 (4)	
C6	0.7210 (4)	0.25601 (9)	0.4274 (3)	0.0177 (4)	
H6A	0.8545	0.2793	0.4849	0.021*	
C1	0.4915 (4)	0.28237 (8)	0.3480 (2)	0.0151 (4)	
C7	0.1530 (4)	0.44583 (9)	0.1445 (2)	0.0154 (4)	
C5	0.7508 (4)	0.19480 (9)	0.4208 (3)	0.0179 (4)	
H5A	0.9047	0.1770	0.4730	0.021*	
C2	0.2936 (4)	0.24722 (9)	0.2616 (2)	0.0184 (4)	
H2A	0.1409	0.2648	0.2068	0.022*	

### Atomic displacement parameters (Å^2^)

**Table:**

	*U*^11^	*U*^22^	*U*^33^	*U*^12^	*U*^13^	*U*^23^
O5	0.0184 (7)	0.0138 (7)	0.0239 (7)	0.0005 (6)	−0.0019 (6)	−0.0006 (6)
O3	0.0210 (7)	0.0179 (8)	0.0173 (7)	−0.0017 (6)	0.0030 (6)	0.0005 (5)
O2	0.0196 (7)	0.0195 (8)	0.0159 (7)	−0.0006 (6)	0.0030 (5)	−0.0005 (5)
O1	0.0163 (7)	0.0186 (8)	0.0197 (7)	−0.0017 (6)	0.0003 (5)	0.0003 (6)
O4	0.0172 (7)	0.0171 (7)	0.0187 (7)	−0.0012 (6)	−0.0032 (5)	−0.0031 (6)
C4	0.0208 (10)	0.0125 (10)	0.0146 (9)	−0.0015 (8)	0.0072 (8)	−0.0004 (7)
C3	0.0204 (10)	0.0195 (11)	0.0170 (10)	−0.0026 (8)	−0.0006 (8)	−0.0034 (8)
N1	0.0257 (9)	0.0178 (9)	0.0150 (8)	−0.0002 (8)	0.0047 (7)	0.0004 (7)
C8	0.0152 (9)	0.0210 (11)	0.0146 (9)	−0.0018 (8)	0.0002 (7)	−0.0008 (8)
C9	0.0160 (10)	0.0111 (9)	0.0175 (9)	0.0026 (8)	−0.0006 (7)	−0.0013 (7)
C6	0.0173 (10)	0.0163 (10)	0.0185 (10)	−0.0016 (8)	−0.0012 (8)	−0.0023 (8)
C1	0.0192 (10)	0.0130 (10)	0.0131 (9)	0.0000 (8)	0.0024 (7)	0.0001 (7)
C7	0.0153 (9)	0.0115 (9)	0.0183 (9)	0.0035 (8)	−0.0010 (7)	0.0015 (7)
C5	0.0156 (9)	0.0200 (11)	0.0173 (9)	0.0018 (8)	−0.0005 (7)	0.0024 (8)
C2	0.0165 (9)	0.0206 (11)	0.0169 (10)	0.0023 (8)	−0.0016 (8)	0.0007 (8)

### Geometric parameters (Å, º)

**Table:**

O5—C1	1.365 (2)	N1—H1B	0.8900
O5—H5	0.8200	N1—H1C	0.8900
O3—C9	1.234 (2)	C8—C7	1.513 (3)
O2—C7	1.248 (2)	C8—C9	1.513 (3)
O1—C7	1.280 (2)	C8—H8A	0.9700
O1—H1	0.8207	C8—H8B	0.9700
O4—C9	1.302 (2)	C6—C1	1.390 (3)
C4—C5	1.378 (3)	C6—C5	1.391 (3)
C4—C3	1.384 (3)	C6—H6A	0.9300
C4—N1	1.473 (2)	C1—C2	1.390 (3)
C3—C2	1.379 (3)	C5—H5A	0.9300
C3—H3A	0.9300	C2—H2A	0.9300
N1—H1A	0.8900		
			
C1—O5—H5	105.8	H8A—C8—H8B	107.2
C7—O1—H1	102.4	O3—C9—O4	123.22 (17)
C5—C4—C3	120.89 (18)	O3—C9—C8	119.73 (17)
C5—C4—N1	120.19 (18)	O4—C9—C8	117.06 (17)
C3—C4—N1	118.91 (17)	C1—C6—C5	119.96 (18)
C2—C3—C4	119.50 (18)	C1—C6—H6A	120.0
C2—C3—H3A	120.3	C5—C6—H6A	120.0
C4—C3—H3A	120.3	O5—C1—C6	123.16 (17)
C4—N1—H1A	109.5	O5—C1—C2	117.24 (17)
C4—N1—H1B	109.5	C6—C1—C2	119.59 (18)
H1A—N1—H1B	109.5	O2—C7—O1	123.55 (18)
C4—N1—H1C	109.5	O2—C7—C8	119.03 (17)
H1A—N1—H1C	109.5	O1—C7—C8	117.41 (17)
H1B—N1—H1C	109.5	C4—C5—C6	119.60 (18)
C7—C8—C9	117.20 (16)	C4—C5—H5A	120.2
C7—C8—H8A	108.0	C6—C5—H5A	120.2
C9—C8—H8A	108.0	C3—C2—C1	120.44 (18)
C7—C8—H8B	108.0	C3—C2—H2A	119.8
C9—C8—H8B	108.0	C1—C2—H2A	119.8
			
C5—C4—C3—C2	−0.2 (3)	C9—C8—C7—O1	−19.3 (3)
N1—C4—C3—C2	178.95 (17)	C3—C4—C5—C6	0.8 (3)
C7—C8—C9—O3	−166.29 (18)	N1—C4—C5—C6	−178.33 (17)
C7—C8—C9—O4	14.4 (3)	C1—C6—C5—C4	−0.5 (3)
C5—C6—C1—O5	179.28 (18)	C4—C3—C2—C1	−0.7 (3)
C5—C6—C1—C2	−0.3 (3)	O5—C1—C2—C3	−178.69 (18)
C9—C8—C7—O2	161.91 (18)	C6—C1—C2—C3	1.0 (3)

### Hydrogen-bond geometry (Å, º)

**Table:**

*D*—H···*A*	*D*—H	H···*A*	*D*···*A*	*D*—H···*A*
O5—H5···O3^i^	0.82	1.94	2.745 (2)	168
N1—H1*A*···O2^ii^	0.89	2.10	2.989 (2)	177
N1—H1*B*···O4^iii^	0.89	2.33	3.090 (2)	144
N1—H1*C*···O2^iv^	0.89	1.98	2.836 (2)	160
O1—H1···O4	0.82	1.65	2.450 (2)	165
C3—H3*A*···O3^iii^	0.93	2.47	3.398 (3)	175
C8—H8*B*···O1^v^	0.97	2.31	3.163 (3)	147

Symmetry codes: (i) *x*+1, *y*, *z*; (ii) *x*+1, −*y*+1/2, *z*+1/2; (iii) *x*, −*y*+1/2, *z*−1/2; (iv) *x*, −*y*+1/2, *z*+1/2; (v) *x*−1, *y*, *z*.
